# Living with an artificial eye: qualitative insights into patient and family member experiences

**DOI:** 10.1007/s10792-024-02933-0

**Published:** 2024-05-22

**Authors:** Florien W. Boele, Jessica Charlotte Kawalek, Emma Nicklin, Taras Gout, Judith M. Watson, The PERSONAL-EYE-S Study Group, Amie Woodward, Elizabeth Coleman, Sarah Ronaldson, Tim Zoltie, Paul Bartlett, Laura Wilson, Emma Walshaw, Tom Archer, Bernard Chang, George Kalantzis, Nabil El-Hindy, Mike Theaker

**Affiliations:** 1 Leeds Institute of Medical Research at St James’s, St James’s University Hospital, University of Leeds, Leeds, LS9 7TF UK; 2 Department of Ophthalmology, St James’s University Teaching Hospital, Beckett St., Leeds, LS9 7TF UK; 3 York Trials Unit, Department of Health Sciences, University of York, York, YO10 5DD UK; 4 Department of Health Sciences, Faculty of Science, The University of York. York Trials Unit, York, UK; 5 School of Dentistry, Faculty of Medicine and Health, University of Leeds, Worsley Building, Leeds, LS2 9LU UK; 6 Leeds Artificial Eye Service, Leeds Dental Institute, Leeds Teaching Hospitals NHS Trust, Worsley Building, Leeds, LS2 9LU UK; 7 Department of Ophthalmology, Leeds Teaching Hospitals NHS Trust, Leeds, LS9 7TF UK; 8 PPI representative, Daventry, UK

**Keywords:** Eye, Artificial, Ocular prosthesis, Quality-of-life, Interviews, Reflexive thematic analysis

## Abstract

**Purpose:**

Artificial eye users (AEUs) can experience a negative impact on psychological and emotional wellbeing, including reduced social functioning, which may be a consequence of living with one eye removed, and/or of having a prosthetic eye. This may have wider consequences for their families. We aimed to explore what it means to live with a prosthetic eye, for both AEUs and their families—and how any quality of life (QoL) issues impact on their day-to-day functioning.

**Methods:**

A subset of AEUs and their family members taking part in a feasibility randomised controlled trial comparing hand-painted to digitally printed artificial eyes were invited for semi-structured interviews. Transcripts were analysed using reflexive thematic analysis. Qualitative results related to trial participation are covered elsewhere. Here, we focus on QoL and day-to-day functioning.

**Results:**

Twelve AEUs (eight males) and five spouses (one male) who had worn artificial eyes for 2–65 years took part, and four themes were identified. (1) Impact on day-to-day life: AEUs and their spouses have to adapt to (partial) sight loss, reduced levels of confidence, and social withdrawal. (2) Impact on psychological and emotional wellbeing: distress among AEUs and their spouses can be severe and prolonged, highlight unmet support needs. (3) Challenges with treatment experiences: AEUs experienced negative impact of fragmentation of care and long waiting times. (4) Worries about the future: AEUs mentioned fragility of remaining sight, and concerns around potential need for further treatment.

**Conclusion:**

Patients and their family members experience negative impact of being an AEU on their everyday lives and quality of life. There is a potential role for psychosocial support services in supporting AEUs and their families even long after eye loss.

## Introduction

Surgical removal of a blind, painful eye may be necessary following severe trauma or tumours [1, 2]. Once the eye socket has healed, a prosthetic eye can be made and fitted. This usually requires replacement every 2–6 years for adults. It is estimated that around 8 million individuals require a prosthetic eye worldwide, and in the UK, there are around 48,000 artificial eye users (AEUs) [3].

AEUs can experience a negative impact on psychological and emotional wellbeing, including heightened levels of anxiety and depression [4–6]. While some recovery has been reported over time [7], issues may persist. Early following eye loss, but also in the longer term (> 2 years later), AEUs are known to suffer from concerns about discharge, visual perception, and appearance, with a loss of self-image [8]. A survey among 217 experienced AEUs (> 2 years) demonstrated negative consequences on recreational, occupational, and social aspects of life [9]. Looking at the very long term, a large sample of adult survivors of retinoblastoma who had undergone enucleation (*N* = 404) reported persistent physical, intrapersonal, social, relational, and affective problems, at a mean of 42 years after diagnosis [10]. Therefore, AEUs are at risk for adverse impact on their quality of life (QoL).

This impacts not just on AEUs themselves but may have consequences for their families as well. While the difficulties encountered by parents of children who have suffered eye loss have been reported previously [11, 12], experiences of family members of adult AEUs are less well-described. Existing literature holds strengths in quantitative data collection through physical assessments, and self- and proxy-reported questionnaires, but these reports may only partially capture what it means to live with a prosthetic eye, for both AEUs and their families—and how any QoL issues impact on their day-to-day functioning. The present article covers qualitative findings from a feasibility trial [13], with a focus on the personal experiences of both AEUs and their family members.

## Methods

### Study design

The parent study is a cross-over feasibility randomised controlled trial (RCT) aiming to evaluate hand-painted and digitally printed artificial eyes, to assess the practicality and usefulness of carrying out a future larger RCT (ISRCTN85921622) [13]. In brief, AEUs received two artificial eyes (order determined by randomisation) and trialled each for two weeks. A subsample took part in in-depth semi-structured interviews to better understand participants’ experiences. Qualitative results related specifically to participation in the trial are covered elsewhere [14], with the current article focusing on AEU and their family members’ quality of life and day-to-day functioning.

The consolidated criteria for reporting qualitative research (COREQ) were used [15]. The study was approved by North-West-Haydock Research Ethics Committee (21/NW/0150).

### Participants

Participants were recruited via clinic, database screening, or adverts on the Royal National Institute of Blind People and Blind Veterans UK websites. Patients were provided with an information pack and if interested, they could return a contact form after which the study team arranged eligibility assessment. Adult AEUs (≥ 18) were eligible if they had worn an artificial eye for over 12 months, were in need of a replacement, and were able to complete study procedures in English. Exclusion criteria were: ongoing clinical concerns such as poor eye socket healing, extrusion, dehiscence; bilateral AEUs; pregnant AEUs, or persons currently shielding (related to the COVID-19 pandemic). Eligible AEUs were invited to name a close contact (CC; e.g. friend, family member, spouse). AEUs could take part without a close contact but not vice versa. All participants provided written informed consent. Following the last clinic appointment related to the trial, participants were booked in for interview with selection based on willingness/availability.

### Data collection

Interviews were performed by an experienced qualitative research assistant (JK, PhD), supervised by senior researchers (FWB, PhD and EN, PhD), none of whom were involved in patient care. Semi-structured interview guides allowed participants to express their views about the impact of their treatments on QoL [16]. First, we covered background information through open questions (e.g. “Could you tell me a little about yourself? What do you like to do?”). Then, we covered participants’ experiences related to being an artificial eye wearer (e.g. “Could you tell me about your experiences as an AEU? What have your treatment experiences been like?”). Impact on everyday activities and quality of life was discussed (e.g. “How have these experiences influenced your quality of life?”). Specific questions about trial participation followed, which are covered elsewhere [14]. For close contacts, questions covered both their view of patient experiences as well as their own. Interviews (1:1) were performed face-to-face at Leeds General Infirmary, by telephone or video call (audio-recorded). Reflexive notes were written.

### Data analysis

Interview recordings were transcribed verbatim. Reflexive thematic analysis, led by JK supervised by EN, was used to develop knowledge based on patterns within the data [17–20]. Data from AEUs and their close contacts were analysed separately and then key themes developed across the groups. For each group (AEU and CC), analysis began with reading transcripts to gain familiarity with the data. Second, the transcripts were coded using NVivo software by labelling small sections of the transcripts with a brief description. This process was repeated so that descriptive labels could be edited to take into account additional information. Codes were modified and organised into subthemes or merged with other codes. Finally, the subthemes and remaining coded data were grouped into themes. An experiential approach (where analysis is grounded in participants’ lived experiences) was used [21].

Investigator triangulation [22] was achieved in the current study by having continuous discussions about study findings with multi-disciplined researchers (FWB and EN) and double-coding 20% of the interviews. Disagreements were resolved through discussion and/or re-examination of transcripts. Finally, one coder (FWB) examined all transcripts again to ensure that the analytical process was robust.

## Results

In total, thirteen AEUs and five of their close contacts were approached for interviews and agreed to take part. One person withdrew due to time constraints. All close contacts were spouses or partners to AEUs. In total, between April and July 2022, twelve AEUs and five close contacts were interviewed, for on average 31 min (range 20–59 min). See Table 1 for participant characteristics.

**Table 1:** Participant demographics

	Artificial eye users (*N* = 12)	Close contacts (*N* = 5)
*Sex N (%)*		
Male	8 (66.7%)	1 (20.0%)
Female	4 (33.3%)	4 (80.0%)
Age (years) median, range	56, 32–80	53, 28–64
*Self-reported ethnicity N (%)*		
White British	10 (83.3%)	5 (100.0%)
British: half Arabic, half English	1 (8.3%)	0 (0%)
Senegalese	1 (8.3%)	0 (0%)
*Cause of eye loss N (%)*		
Accident	7 (58.3%)	N/a
Medical condition	3 (25.0%)	N/a
Cancer	2 (16.7%)	N/a
Time since eye was lost (years) Median, range	9.5, 2–65	N/a

N/A not applicable

**Fig.1:**
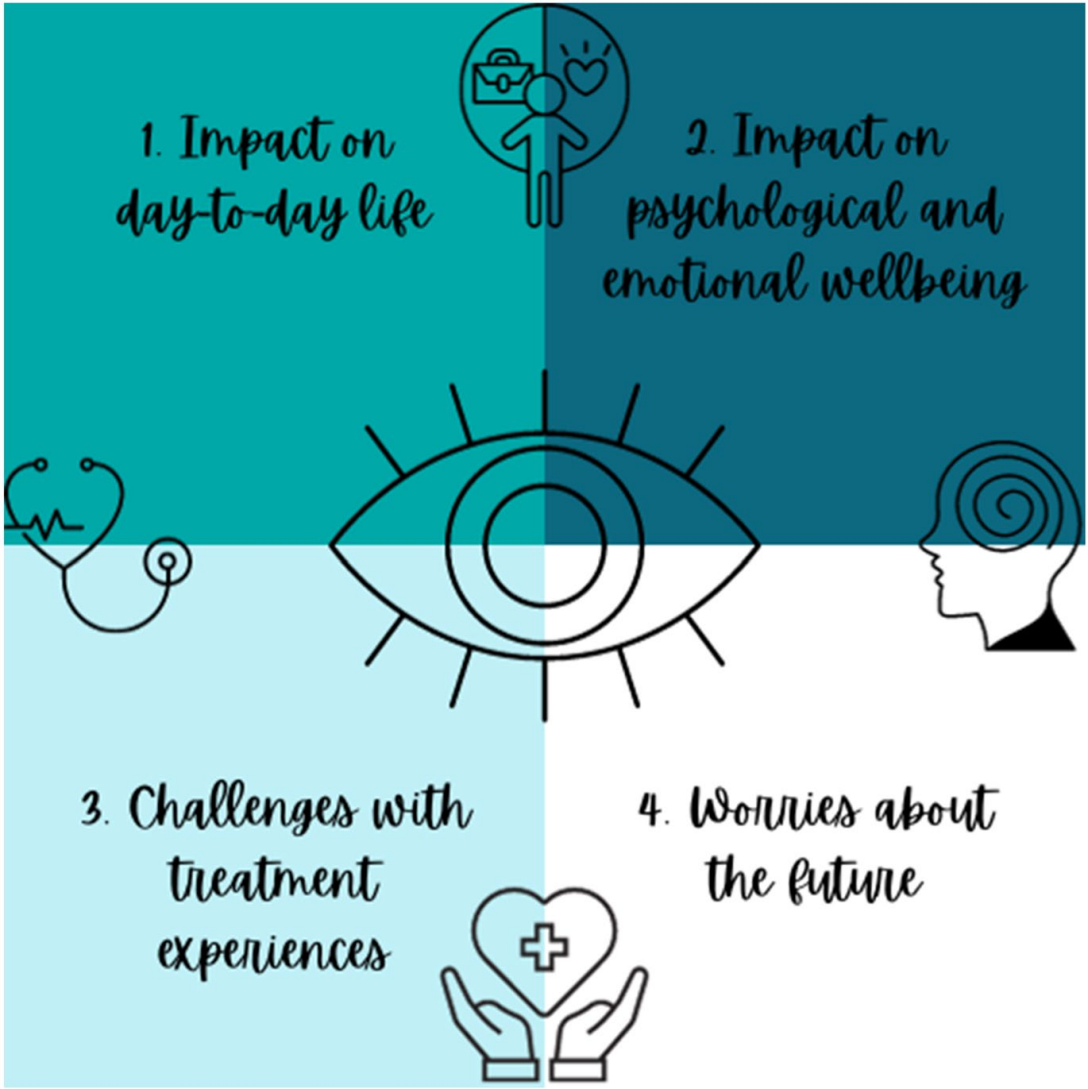
Depiction of the four main themes

Four themes were identified across groups: (1) Impact on day-to-day life; (2) Impact on psychological and emotional wellbeing; (3) Challenges with treatment experiences; (4) Worries about the future (Fig. 1).

### Impact on day-to-day life

Being an AEU typically negatively impacts on day-to-day living, although the extent to which this bothers people can vary. Just one participant, who suffered a long-standing medical condition prior to having his eye removed, reported only benefits for his daily life from getting a prosthetic eye. In most interviews, having to regain confidence is frequently mentioned. The eye loss can cause facial disfigurement as well as impaired vision:

I mean it’s still the eye loss because if I’m not in a familiar environment I still bang into things. I used to fall down a lot initially which is really embarrassing because it makes you look like you’re drunk and you’re not drunk but you’re falling because you’re having to adjust to the fact you've only got like single vision. [Female AEU, 51, accident].

Apart from worrying about other people’s judgement, some participants described how even simple everyday tasks like putting toothpaste on a brush or lining a screwdriver up with a screw could be a struggle:

And if I’m doing something that’s really intricate, I start shaking like me nerves have gone, it’s, I tremble because there’s that much concentration needed to try and solve it. [Male AEU, 61, accident].

Family members describe supporting AEUs as best they can with everyday activities such as driving, tidying up, and cooking:

Whether I’ve been as supportive as she would have liked you would have to ask her but I certainly do a lot, I’ve always done a lot of the cooking. We’ve always been a good team and that is still the case. [Male CC, 53].

Many AEUs described reduced social activities including avoiding crowded situations, being reluctant to make new friends and feeling self-conscious: “*you’re always conscious people are staring and looking*” [Male AEU, 63, accident]. This can have a big impact on partners as well: “*Socially we don’t, we don’t go out, we don’t socialise with friends or anything since he lost his eye*” [Female CC, 51].

Others admitted to losing their confidence in social situations, due to their loss of vision, their altered appearance, and relying on family members or friends for practical and emotional support. Social decline seemed most prevalent closer to eye loss: *“After my accident, I was home a lot and it just really got me down”* [Male AEU, 32, accident]. For some AEUs it had taken several years to feel more socially confident:

First 5, 6, 7 years was really a bit of a strain…if you were invited anywhere, you didn’t want to be seen, you sort of become a bit of a hermit [Male AEU, 63, accident].

To some extent this appeared linked to reduced ability to drive:

I go out because I have an appointment or I need to go to hospital or to see the dentist, like that, the important things I will do, I will go out but now…But not socially? No. [Male AEU, 45, medical condition].

Yet, AEUs expressed determination to carry on with daily life as best they could: “*I just decided there’s nothing I can do, it’s not going to come back, I’ve lost it, and I just get on with it*” [Male AEU, 69, accident], with many focusing on positive thoughts:

I have to be confident, that’s the thing, as confident as I possibly can be anyway, but yeah, it’s just, it’s all about trying, I’m trying to get on with life in general really so yeah, you have to be confident. And show, basically try to show as little emotion as possible around, you know, I’m not going to go around moping that I had a ridiculous accident sort of 4/5 years ago, but you’ve just got to get on with it. [Male AEU, 61, accident].

At the time of eye loss AEUs reported having extended time off work to recover. For some AEUs, returning to their work was impossible, especially if their job included driving, meaning they had to try and establish another career:

The accident with my eye has rendered me unable to do the work I was doing…we’re just plodding on now trying to make a living as best we can, doing whatever I can do [Male AEU, 61, accident].

### Impact on psychological and emotional wellbeing

After eye removal, many AEUs suffered from anxiety, depression, post-traumatic stress disorder and anxiety-induced insomnia. In some cases, psychological problems were severe and lasted for years, with both AEUs and some of their family members seeking professional mental health support. Family members sometimes felt more formal support was necessary: *“[AEU] has had a couple of counselling sessions…he hasn’t seen anybody, it’s just literally telephone calls, so I think he needs to sit with somebody”* [Female CC, 28].

AEUs reported they struggled to adjust to their changed appearance. Some avoided looking in the mirror. Even if the look of their eye had generally improved over time, many were still unhappy with their appearance:

Your life is immediately changed! I still think it looks dreadful but if you’d seen pictures of it initially, obviously it was like many times worse [Female AEU, 51, accident].

A family member described how the AEU’s lack of confidence prevents them from taking out their eye when it gets uncomfortable, even around their family:

When he’s tired and he wants to take it out, he’s still a bit… He doesn’t like the idea of not having an eye in. [Female CC, 60].

AEUs who lost their eye due to trauma as adults reported worse mental health issues. One AEU said the only thing that helped her move on was the improvement of her artificial eye: “*Realising things are actually moving on…I felt a lot better.*” [Male AEU, 32, accident]. But AEUs who lost their eye at a young age said they were able to lead ‘normal’ life because they had never known any different: “*I’ve had the same eye since I was 6 months old…this is me*” [Female AEU, 41, cancer].

While not all family members reported experiencing psychological or emotional issues themselves, some did struggle with feelings of sadness and frustration when their relative lost an eye. One spouse said: “*He’s just not, he’s not same person that he were, he’s really not, really not*” [Female CC, 51]. At times these issues were related to AEUs interlinked cognitive issues:

Because she’s had a brain injury at the same time as the kick, she loses her memory. Her short-term memory has changed and some of the stuff that I’d assumed she’d remember that she did years ago, she doesn’t always remember. So I find that difficult because all those memories that we shared she can’t remember I find it difficult, and I find that really odd. [Male CC, 53].

### Challenges with treatment experiences

In discussing AEUs treatment experiences, many expressed having experienced issues due to fragmentation of care, with a lack of collaboration between different service providers:

They were completely separate entities fighting against each other…rather than working together for the best outcome. [Male CC, 53].

Building a trusted relationship with healthcare providers was found important, with one participant describing a change in treating consultant made her feel vulnerable:

It’s building relationships all over again. I did struggle with that a bit really because I was only ever used to one consultant so I was very conscious about it, so again, being in that environment and showing people who there’s only been a handful in my life that has seen me without it. [Female AEU, 41, cancer].

One participant describes a particularly bad administrative failure resulted in losing eyes kept on file:

They kept it in my records, this eye, so that every time they could send it, but someone must have decided in the office that the files were all too big with all the eyes in so they took all the eyes out and threw them away which for me was a completely disaster, I was totally gutted. [Female AEU, 37, medical condition].

Participants found the wait times for hand-painted eyes unacceptably long. Many AEUs described having to wait for five to nine months, having several reiterations before receiving their first artificial eye, and wait times could also be long for replacement eyes. A long-standing AEU describes challenges around losing his prosthetic eye and having to wear an eye patch:

Probably through fault of my own where I’ve had a couple of accidents where I’ve actually lost them and then there’s just the time scape, you know, between… Well, one was pushing 3½ months without a false eye which was, you know, wearing a patch. Because you’ve had it so long and then people don’t know about your accident, they tend to think, oh I had no idea and then obviously you’re wearing an eye patch. [Male AEU, 63, accident].

Long wait times were found to negatively affect AEUs emotional wellbeing and QoL and could impact on important live events:

But we were meant to go away to Mexico in the September of 2021 for our honeymoon and I was like, oh my God he’s not going to get his eye, it’s going to be awful. [Female CC, 28].

Regarding the appearance of a prosthetic eye, AEUs frequently mentioned the importance of a good, realistic colour match to the other eye. A well-centred eye was also found critical to AEUs satisfaction:

Having it centred is actually more important than the colour match… But I would take a centred one that’s the right size over a right colour one that’s the wrong size or it’s not centred. Because those last two things if they’re wrong, they start to increase the ability of other people to recognize that it’s not a real eye whereas when all those three things are right, people that don’t know me don’t realize it’s an artificial eye and that’s like the holy grail of what you’re trying to achieve. [Female AEU, 51, accident].

Family members similarly expressed negative consequences of a poor fit or colour-match. Family members discussed challenges in supporting their AEU relative emotionally while also adapting to their changed appearance: “*I just felt sick, it just scared me*” [Female CC, 60] and trying to protect them from unwanted attention:

I catch myself getting really annoyed at people if they give her a double take… I want to slap them…if there's something not quite right, if it is not centred or something that’ll make people stare and I get offended on her behalf. [Male CC, 53].

Families felt they received little support:

It’s like if you lose an arm you, you get rehabilitation, or if you have a bad break or things like that, and literally he was sent home day after, so he had his accident and he went to theatre they had to remove his eye and next morning he got discharged with paracetamol and just left…we just got left. [Female CC, 51].

### Worries about the future

While participants were experienced AEUs, worries about the future came up regularly in the interviews. With one eye removed, increased vulnerability of the remaining functioning eye was concerning. A participant described worries about her sight issues progressing, causing full blindness:

I’m still really frightened about my other eye because if anything happens to it, as far as I’m concerned my life is over and I mean that because if anything happens to this eye I’ll be completely blind and I just won’t want to live anymore to be honest I just I wouldn’t. [Female AEU, 51, accident].

Some participants were concerned about having to undergo further treatment, and the associated health risks:

I honestly don’t know what’s going to happen because I don’t want any more general anaesthetics and I can’t just keep having general anaesthetics for the rest of my life, they’re quite, you know there is risk associated with them. [Female AEU, 51, accident].

Others mentioned worries about the availability of effective treatment options should issues recur:

Now it’s a bit of a worry that if it droops again in the future whether I can actually have it [treatment] done again because it’s not healed as well as what it did last time. They won’t probably be able to correct it as well. [Female AEU, 37, medical condition].

Costs associated with treatment which would not be covered through the National Health Service, such as non-permanent facial fillers, could be concerning:

I’ve got to try and decide what I’m going to do about that because my medical insurance won’t pay for it and it’s expensive. [Female AEU, 51, accident].

## Discussion

Our qualitative interviews provide insight into the lived experiences of AEUs and their spouses. Being an AEU impacts on everyday life and can negatively affect wellbeing, often for many years. Social withdrawal was common due to AEUs’ appearance-related anxieties and reduced confidence from partial sight loss. Previous quantitative studies already showed that staring from members of the public can cause emotional struggles in AEUs [4, 5], which was confirmed in our interviews. Conversely, better appearance of the prosthetic eye appears linked to a positive impact on social interactions [23]. But over and above this, we note there is a profound psychological and emotional impact from being an AEU. Distress can be severe and prolonged. These issues were especially prevalent amongst AEUs who lost their eye due to trauma as adults, and those closer to eye removal. Previous quantitative evidence already highlighted that a significant proportion of AEUs suffers from heightened levels of distress [6, 24]. In one quantitative study in 39 patients, no relationship between psychological wellbeing and age of acquisition of the prosthetic, or duration of wear was found [6]—whereas we observed a trend for those closer to eye removal suffering greater distress. This discrepancy highlights that further research is needed to unpick the factors associated with higher distress, so we can offer timely support to AEUs who might benefit from it.

We observed that the everyday impact and psychological and emotional issues related to being an AEU also impacts on relatives. Elevated levels of distress and a need for more support were expressed by our participants. Some mentioned also being bothered by judgement of others, others reported their loved one’s reduced confidence levels affected their relationship and wider social life. It should be noted that we only interviewed a small number of spouses, introducing a potential bias. We have not been able to find publications on the impact of eye loss on family members, other than parent perspectives. While these parental caregivers of children who lose an eye also report emotional difficulties [11, 12], their experiences are different in that their child is fully dependent upon them, with parents advocating on their behalf rather than alongside the patient in a spousal relationship.

AEUs treatment experiences were negatively influenced by fragmentation of care, and long waiting times for a well-matching first or replacement artificial eyes. In some cases, lack of continuity and administrative failure played a role in reduced satisfaction. Worries about the future included the fragility of the remaining functioning eye with potential for complete sight loss, and the need for, and availability, effectiveness, and costs of any further treatment. Previous work on satisfaction of prosthetic eye wearers tends to focus on (the impact of) clinical variables and symptoms [e.g. 23, 25–27. This qualitative exploration shows that there are other factors that are of importance to both AEUs, and their family members.

This study holds strengths in its qualitative exploration of personal experiences of AEUs and their spouses. Limitations include sampling from a single site, potentially limiting applicability of findings to other services; the smaller number of family members who took part, leading to potential bias in views expressed; and the fact that this work took place during the COVID-19 pandemic, which may have impacted on who took part in interviews. While further research is necessary, this work suggests that AEUs and their families typically have unmet rehabilitative and supportive care needs, which would benefit from addressing even long after eye removal.

## Data Availability

All raw data are held securely by the research team and access can be requested if required.
